# IL1RN and KRT13 Expression in Bladder Cancer: Association with Pathologic Characteristics and Smoking Status

**DOI:** 10.1155/2014/184602

**Published:** 2014-07-08

**Authors:** Thomas S. Worst, Verena Reiner, Ute Gabriel, Christel Weiß, Philipp Erben, Thomas Martini, Christian Bolenz

**Affiliations:** ^1^Department of Urology, Mannheim Medical Center, University of Heidelberg, Theodor-Kutzer-Ufer 1–3, 68167 Mannheim, Germany; ^2^Department for Statistical Analysis, Mannheim Medical Center, University of Heidelberg, Mannheim, Germany

## Abstract

*Purpose*. To validate microarray data on cytokeratin 13 (KRT13) and interleukin-1 receptor antagonist (IL1RN) expression in urothelial carcinoma of the urinary bladder (UCB) and to correlate our findings with pathologic characteristics and tobacco smoking. *Methods*. UCB tissue samples (*n* = 109) and control samples (*n* = 14) were obtained from transurethral resection and radical cystectomy specimens. Immunohistochemical staining of KRT13 and IL1RN was performed and semiquantitative expression scores were assessed. Smoking status was evaluated using a standardized questionnaire. Expression scores were correlated with pathologic characteristics (tumor stage and grade) and with smoking status. *Results*. Loss of KRT13 and IL1RN expression was observed in UCB tissue samples when compared to controls (*P* = 0.007, *P* = 0.008) in which KRT13 and IL1RN expression were high. IL1RN expression was significantly reduced in muscle-invasive tumors (*P* = 0.003). In tissue samples of current smokers, a significant downregulation of IL1RN was found when compared to never smokers (*P* = 0.013). *Conclusion*. Decreased expressions of KRT13 and IL1RN are common features of UCB and are associated with aggressive disease. Tobacco smoking may enhance the loss of IL1RN, indicating an overweight of proinflammatory mediators involved in UCB progression. Further validation of the influence of smoking on IL1RN expression is warranted.

## 1. Introduction

Urothelial carcinoma of the urinary bladder (UCB) is the seventh most prevalent type of cancer worldwide. In the United States, an estimated 73,000 new cases and 14,000 deaths occurred during 2012 [1]. Carcinogenesis of UCB often requires exposure to occupational carcinogens and environmental pollution [2]. Other risk factors include hereditary susceptibility and specific medical treatments [3, 4]. Tobacco smoking has been identified as a major lifestyle risk factor for developing UCB. It has been estimated that around 50% of all UCB cases can be attributed to tobacco smoking, with considerable variation in groups of former and current smokers [5].

Many of the ingredients in tobacco smoke such as aromatic amines are renally excreted and therefore directly expose the urothelium to toxic metabolites [6, 7]. Due to its reservoir function the exposure time is the longest in the urinary bladder. Metabolites of tobacco smoke may cause inflammatory reactions and lead to perturbation of cellular processes which may alter gene expression [8, 9]. We have previously reported on changes of different gene expressions in UCB tissue depending on smoking status in a microarray study [10]. Many of these genes are associated with inflammation processes as well as the integrity of the cytoskeleton and cell structure. Among these cytokeratin 13 (KRT13) and interleukin-1 receptor antagonist (IL1RN) were strongly downregulated in UCB tissue of smokers. KRT13 is a gene encoding for an intermediate cytoskeleton protein and is typically expressed in the basal compartments of stratified epithelial tissues [11]. IL1RN is a member of the interleukin-1 superfamily and functions as an antagonist at the interleukin-1 (IL1) receptors 1 and 2 [12]. Therefore, it is capable of confining inflammatory processes mediated by other interleukins. The role of IL1RN during UCB carcinogenesis has not yet been elucidated.

In order to further evaluate the relevance of these two genes and their products in UCB, we aimed to validate our previous findings on the protein level by immunohistochemical analysis of UCB tissue samples.

## 2. Material and Methods

The procedures described in the present study have been approved by the local ethical review committee (reference number 2007-030 N-MA).

### 2.1. Patient Characteristics and Tissue Samples

Formalin fixed paraffin embedded (FFPE) tissue samples of 109 patients (19 women and 90 men, mean age 61 years, range: 41–94 years) diagnosed with UCB were retrospectively evaluated. Of these tumors 95 were primary UCBs and 14 were recurrent UCBs. Tissue samples were obtained either during transurethral resection (*n* = 73) or during radical cystectomy (*n* = 36). From the latter 14 tumor-free FFPE tissue samples served as controls, resulting in a total of 123 tissue samples. The surgical procedures were performed in one institution. All specimens were routinely classified according to the 2002 TNM classification of the American Joint Committee on Cancer. Tumor grade was assessed according to the 1998 WHO/International Society of Urologic Pathology consensus classification. All patients had answered a standardized questionnaire regarding their smoking behavior. According to this they were classified as current smokers (CS), former smokers (FS), and never smokers (NS).

### 2.2. Immunohistochemistry

Tissue samples were sectioned (4 *μ*m thickness) and cut, placed on glass slides, and dried overnight at 36°C. The next day slides were deparaffinised and rehydrated, followed by a peroxidase block and several washing steps in tris-buffered saline (TBS). Slides were steam-heated with a target retrieval solution and subsequently incubated in ice-cold water. After a further washing step in TBS and a 20 min pretreatment in DAKO Real antibody diluent (DAKO, Glostrup, Denmark) the slides were incubated with mouse monoclonal anti-human KRT13 (Novocastra, Leica Biosystems, Wetzlar, Germany) or rabbit polyclonal anti-human IL1RN (Sigma-Aldrich, St. Louis, MO, USA) primary antibodies for 1 hour at room temperature (anti-KRT13) or for overnight incubation at 4°C (anti-IL1RN). Both antibodies were diluted 1 : 200 in DAKO Real antibody diluent (DAKO, Glostrup, Denmark). After two washing steps in TBS, secondary EnVision HRP-labeled anti-mouse or anti-rabbit antibody (both DAKO, Glostrup, Denmark) was added for 40 min incubation at room temperature. After three further washing steps, visualization was performed with AEC+ substrate chromogen (DAKO, Glostrup, Denmark) as recommended by the manufacturer. Counterstaining was performed with haematoxylin. The slides were coverslipped using Faramount aqueous medium.

### 2.3. Microscopic Evaluation

Microscopic evaluation was performed using a Leica Axioskop 2 Plus (Carl Zeiss, Jena, Germany) under standardized conditions with a 10x eyepiece and a 40x objective. The protein expression status of KRT13 and IL1RN was scored semiquantitatively (− none/very low (≤10%), + low, ++ moderate, and +++ high). Five fields of view were evaluated per slide.

### 2.4. Statistical Analysis

According to tumor stage and grade, samples were classified into group 1 (benign controls), group 2 (PUNLMP and pTa low grade), group 3 (high grade non-muscle-invasive tumors: pTa high grade, pTis, and pT1), and group 4 (muscle-invasive tumors: pT2 and higher). Protein expression of KRT13 and IL1RN was compared between UCB tissue samples (groups 2–4) and controls (group 1). Subsequently, groups 2 (*n* = 45), 3 (*n* = 32), and 4 (*n* = 32) were compared independently with controls (*n* = 14). Multiple correlations between smoking status (CS versus NS and CS/FS versus NS) and protein expression were performed: (1) among all 123 tissue samples (109 UCBs and 14 controls), (2) among 109 UCB tissue samples, and (3) among 14 controls and the corresponding UCB tissue samples from the same patients (*n* = 28). All statistical evaluations were conducted using the Exact Trend Test (SAS software, release 9.3, SAS Institute, Cary, USA). *P* values <0.05 were considered statistically significant.

## 3. Results

Table 1 displays the distribution of UCB stage, grade, and smoking behavior among the patients and tissue samples. Microscopic evaluation showed an almost exclusive cytoplasmic expression of KRT13 in epithelial cells. IL1RN was also associated with the cell membrane and present in the extracellular space. The expression scores and correlations are summarized in Table 2. Considering the entire group of tissue samples, the expression of KRT13 was significantly lower in UCB when compared to controls with no morphologic urothelial alterations (*P* = 0.007).

**Figure 1:**
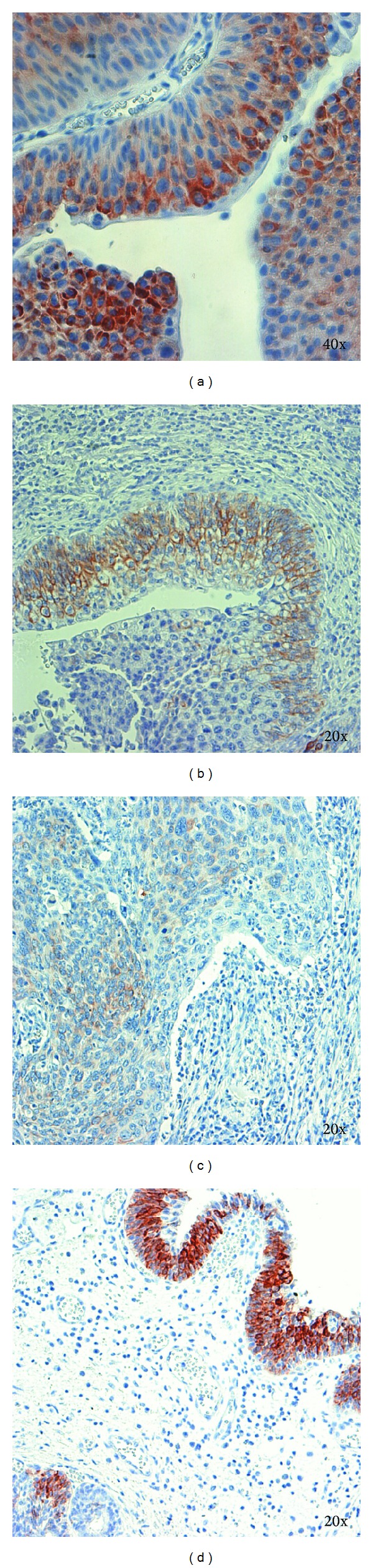
IL1RN expression was in general moderate to high in group 2 ((a) pTa low grade), decreased in group 3 ((b) pT1), and was significantly lower in group 4 ((c) pT3). Benign urothelial tissue (group 1) showed the most intensive staining (d).

The immunoreactivity for IL1RN among all 109 UCB tissue samples was significantly weaker when compared with controls (*P* = 0.008). Furthermore an inverse correlation was seen between an increasing UBC stage and the expression of IL1RN, showing the strongest correlation for the group of muscle-invasive UCB alone (*P* = 0.003). Representative immunohistochemistry images showing the expression of IL1RN in different tumor stages are displayed in Figure 1.

The expression of KRT13 was not significantly associated with smoking status. Similarly, no significant association was found between IL1RN expression and smoking status when considering the entire cohort. However, when focusing on the subgroup of UCB tissue samples and the control samples from the same patients (*n* = 16) of only CS and NS, a significant inverse correlation (*P* = 0.013) between IL1RN expression and smoking status was observed. These correlations between the expression of KRT13 and IL1RN and smoking status are summarized in Table 3.

## 4. Discussion

In the present study we validated our previous findings on altered gene expression of KRT13 and IL1RN in UCB. Potential associations between protein expression status and tobacco smoking were evaluated.

Cytokeratins are cytoskeleton proteins and represent specific markers for epithelial cells of any origin. Several cytokeratins have been identified as prognostic markers in different tumor entities [13, 14]. KRT13 was reported to occur in combination with KRT8, KRT18 and KRT20 in UCB [11]. According to our own data KRT13 is less expressed in UCB. Other studies [15–18] show a downregulation of KRT13 expression in more advanced stages and in high grade UCB. Recent findings suggested an epigenetic alteration of KRT13 gene due to hypermethylation leading to diminished expression of this gene in aggressive UCB [19]. Such correlations are consistent with previous findings in studies on epithelial to mesenchymal transition (EMT) during which dedifferentiation of epithelial tumor cells into a mesenchymal phenotype may occur [20, 21]. During EMT tumor, cells acquire a higher grade of plasticity and become more likely to invade the surrounding tissue and to metastasize [22]. EMT may cause altered protein expression in UCB cells. However the type and number of proteins involved are not well known.

We were unable to show an association between a decreased expression of KRT13 and tobacco smoking which is in discordance with our previous findings [10]. The lack of data about the smoking dependency of KRT13 expression in UCB in the current literature precludes comparisons with other cohorts. Interestingly, the influence of tobacco smoke was associated with the occurrence of KRT13 overexpressing squamous cell metaplasia in the upper airway tract epithelium [23, 24]. Although these data are derived from animal models changes of KRT13 expression in human tissue directly exposed to tobacco smoke itself or to its ingredients, like urothelial tissue, seem to be possible.

We found a significant correlation of decreased IL1RN expression in UCB of current smokers. Its agonistic counterpart IL1 represents a potent mediator of inflammation and its tissue concentrations are elevated in many nonmalignant and malignant diseases [25, 26]. A pharmaceutically engineered analogon of IL1RN has become a treatment option for advanced rheumatoid arthritis and other chronic inflammatory diseases [27, 28]. Genetic alterations and polymorphisms in the IL1RN gene have been associated with an increased risk for developing different malignancies, including gastric cancer [29], prostate cancer [30], and UCB [31, 32]. Therefore, alterations of IL1RN may be involved in the carcinogenesis of UCB. We hypothesize that an impaired function or amount of IL1RN disturbs the equilibrium between pro- and anti-inflammatory factors, leading to an excessive inflammatory stimulus. We found an inverse correlation between tissue protein levels of IL1RN and more advanced tumor stages. Whether a low expression of IL1RN is an early driver of UCB carcinogenesis or a later consequence of complex alterations in advanced tumor stages remains to be clarified in further investigations.

We tested a potential association between exposure to tobacco smoke as a major risk factor for UCB and IL1RN expression in a subgroup of UCB tissue samples and controls from CS and NS. A significant correlation between a decreased expression of IL1RN and smoking was found, suggesting an interference of tobacco smoke ingredients with UCB tissue.

Our study is limited by a relatively small sample size, especially regarding control samples and in the last named subgroup of CS and NS. Also the study uses benign tissue from UCB patients and not tissue samples from healthy individuals as controls. Therefore a bias of tumor-associated effects in control samples cannot be excluded. The use of immunohistochemistry is well established; however, it has inherent problems, for example, variable antibody choice and interpretation criteria as well as a potential inconsistency in specimen handling and technical procedures. However, immunohistochemical staining was an adequate validation method to test the protein expression status in the present study.

## 5. Conclusion

We confirmed a decreased protein expression of KRT13 in UCB as a potential indicator of EMT and increased cell plasticity. Decreased expressions of both KRT13 and IL1RN are common features of UCB and are associated with aggressive disease. In a subgroup of samples and controls IL1RN was significantly lower expressed in UCB of current smokers. Tobacco smoking may enhance the loss of IL1RN protein, potentially indicating an overweight of proinflammatory mediators involved in UCB progression. Further validation of the influence of smoking on IL1RN expression is warranted. Therefore transcriptomic profiling of other pro- and anti-inflammatory genes both on the protein and on the mRNA level should be performed to better understand the kinetics of inflammatory processes in UCB carcinogenesis.

## Figures and Tables

**Table 1:** Pathologic and smoking characteristics of 109 urothelial carcinoma of the bladder samples and 14 controls.

Group	Stage	*n*	NS	FS	CS
1 (controls)	*n*	**14**	6	6	2

2 (low grade)	PUNLMP	5	1	3	1
	pTa low grade	40	15	18	7
	*n*	**45**	16	21	8

3 (high grade—non-muscle-invasive)	pTa high grade	7	3	2	2
	pTis	2	1	0	1
	pT1	23	10	6	7
	*n*	**32**	14	8	10

4 (muscle-invasive)	pT2	20	3	10	7
	pT3	9	5	4	0
	pT4	3	0	2	1
	*n*	**32**	8	16	8

**2–4** (UBC samples)	**n**	**109**	**38**	**45**	**26**

**1–4** (all samples)	**n**	**123**	**44**	**51**	**28**

CS = current smoker; FS = former smoker; NS = never smoker; PUNLMP = papillary urothelial neoplasm of low malignant potential.

**Table 2:** Correlations between KRT13 and IL1RN expression and tumor characteristics in UCB tissue samples compared with controls.

CK13	Group 1 (*n* = 14)	Group 2 (*n* = 45)	Group 3 (*n* = 32)	Group 4 (*n* = 32)	Groups 2–4 (*n* = 109)
− (≤10%)	4 (28,6%)	27 (60,0%)	17 (53,1%)	19 (59,4%)	63 (57,8%)
+	4 (28,6%)	9 (20,0%)	9 (28,1%)	9 (28,1%)	27 (24,8%)
++	1 (7,1%)	5 (11,1%)	3 (9,4%)	1 (3,1%)	9 (8,3%)
+++	5 (35,8%)	4 (8,9%)	3 (9,4%)	3 (9,4%)	10 (9,2%)

*P* value	Reference	0.019	0.045	0.015	0.007

IL1RN					

− (≤10%)	0 (0,0%)	10 (22,2%)	8 (25,0%)	9 (28,1%)	27 (24,8%)
+	0 (0,0%)	5 (11,1%)	4 (12,5%)	5 (15,6%)	14 (12,8%)
++	4 (28,6%)	9 (20,0%)	7 (21,9%)	7 (21,9%)	23 (21,1%)
+++	10 (71,4%)	21 (46,7%)	13 (40,6%)	11 (34,4%)	45 (41,3%)

*P* value	Reference	0.020	0.010	0.003	0.008

Group 1 = benign tissue; group 2 = low grade tumors; group 3 = high grade UCB (non-muscle-invasive); group 4 = muscle-invasive UCB.

**Table 3:** Correlations between KRT13 and IL1RN expression and smoking status among all samples and a subset from patients providing both tumor and control samples (only CS and NS were considered).

Protein	Samples	Smoking	−	+	++	+++	*P* value
KRT13	All (72)	NS	26	10	5	3	0.395
		CS	11	9	4	4	
	Tumors and benign (16)	NS	4	5	2	1	0.384
		CS	0	2	2	0	

IL1RN	All (72)	NS	8	4	12	20	0.695
		CS	4	5	6	13	
	Tumors and benign (16)	NS	0	1	3	8	0.013
		CS	2	0	2	0	
